# Prognostic value of clinical and radiological findings for conservative treatment of idiopathic ulnar impaction syndrome

**DOI:** 10.1038/s41598-018-28060-2

**Published:** 2018-06-29

**Authors:** Young Hak Roh, Sangwoo Kim, Hyun Sik Gong, Goo Hyun Baek

**Affiliations:** 1 Department of Orthopaedic Surgery, Ewha Womans University Medical Center, Ewha Womans University College of Medicine, 1071 Anyangcheon-ro, Yangcheon-gu, Seoul, 07985 South Korea; 2 Department of Orthopaedic Surgery, Seoul National University College of Medicine, Seoul National University Bundang Hospital, 173 Gumi-ro, Bundang-gu, Sungnam, 13620 South Korea; 3 Department of Orthopaedic Surgery, Seoul National University College of Medicine, Seoul National University Hospital, 101 Daehak-ro, Jongno-gu, Seoul, 03080 South Korea

## Abstract

Ulnar impaction syndrome (UIS) is a common source of ulnar-sided wrist pain, yet not all cases of radiographic ulnar impaction are symptomatic. We retrospectively analyze clinical or radiologic factors that affect prognoses of conservative treatment for idiopathic UIS. A total of 114 patients who had been diagnosed with UIS were treated with 6 weeks of short arm orthosis followed by formal physiotherapy for 6 weeks, with lifestyle modification to limit aggravating movements. The response to treatment, including pain numeric rating scale on an ulnar provocation test, grip strength, Disability of the Arm, Shoulder, and Hand score (DASH), was assessed at 24-week follow-up. For the 24-week follow-up, 29 patients (25%) underwent ulnar shortening osteotomy due to persistent symptoms after conservative treatment, and 18 (16%) patients had pain scores of greater than 5, but they had not undergone surgery. After controlling for confounding variables, female gender (odds ratio (OR) 1.39), duration of symptom (OR 1.27), high pain NRS score on provocation test (OR 1.45), and enhanced carpal or distal ulna bone on MRI (OR 1.82) were associated with a higher likelihood of treatment failure. Knowledge of the factors offers physicians insight into predicting prognoses and helps patients set realistic expectations.

## Introduction

Ulnar impaction syndrome (UIS) is a common source of ulnar-sided wrist pain^1^. It is a degenerative condition that occurs secondary to excessive load across the ulno-carpal joint^2^. The condition is termed idiopathic UIS when patients have congenital or dynamic positive ulnar variance with wrist pronation and forceful grip without any history of fracture or premature physeal arrest^3^. Patients complain of ulnar-sided wrist pain and occasionally swelling and loss of wrist motion and forearm rotation^4,5^. Although this condition is usually associated with positive ulnar variance, it is not unusual to see a patient with radiographic evidence of ulnar impaction but who has minimal or no symptoms, and not all cases of ulnar impaction are symptomatic^6^. Furthermore, there is little information known about the prognostic factors and natural history of UIS^7^.

Radiologic evaluations including magnetic resonance imaging (MRI) are frequently performed for patients with UIS because it allows for earlier detection of an abnormality in the triangular fibrocartilage complex (TFCC), cartilage, or bone marrow of carpal bones; it is also helpful in extensive differential diagnosis for patients with ulnar-sided wrist pain^8–10^. However, few studies have investigated the correlations between radiologic findings and the clinical symptom severity and prognosis of UIS. Clarity regarding these radiologic findings and their correlations with the clinical symptoms and prognoses would be useful for individualizing and quantifying each patient’s condition.

Little information is currently available to analyze unsatisfactory outcomes after conservative treatment for idiopathic UIS, and knowledge of these factors will offer physicians insight into predicting prognoses and help determine the proper indication for surgical treatment for UIS. The purpose of this study was to analyze the factors that affect clinical symptoms and prognoses of conservative treatment for UIS. We hypothesized that certain demographic, clinical, and radiologic features would be associated with unsatisfactory outcomes.

## Materials and Methods

A total of 198 patients diagnosed with UIS at an urban tertiary referral hospital between June 2013 and May 2015 were retrospectively enrolled in this study; the diagnosis had been based on clinical symptoms and physical examination with radiographic confirmation of positive ulnar variance. The inclusion criteria for the diagnosis were (1) a history of ulnar wrist pain that worsened with pronation and ulnar deviation of the wrist during such activities as opening a jar, squeezing a wet towel, typing, or changing the gearshift in an automobile for more than three months; (2) no history of trauma of the forearm and wrist; (3) a positive provocation test (ulno-carpal stress test); and (4) plain radiograph with positive ulnar variance in neutral or pronation power-grip position. Patients underwent complete wrist examinations to rule out other sources of pain such as pisotriquetral arthritis, distal radioulnar joint arthrosis, extensor carpi ulnaris subluxation or tendonitis, or neuritis of the dorsal cutaneous branch of the ulnar nerve. The exclusion criteria were radiographic evidence of an old fracture of the forearm or wrist or radiographic evidence of congenital anomalies such as a Madelung deformity; based on these, 181 patients were approached for study. Of those, 123 patients had undergone MRI to identify abnormality in the TFCC, cartilage, or bone marrow of carpal bones. Among these patients, nine (7%) were lost to follow-up before six months, leaving 114 for analysis here (Table 1). This study was approved by the institutional review boards of Gil Medical Center, and all participants provided written informed consent. This study did not required any deviation of the current clinical practice and was conducted in accordance with the principles of research involving human subjects as expressed in the Declaration of Helsinki (64th, 2013) and with Good Clinical Practice standard.

**Table 1:** Clinical and radiographic characteristics of participants.

Characteristics	Number or Score
Participants	114
Mean age (years)	40 (20–58)
Male/female	29 (25%)/85 (75%)
Body mass index (kg/m^2^)	27.8 (18.8–34.2)
Less than a high school education	25 (22%)
Heavy manual labor/clerical with repetitive work/others (including unemployed)	23/41/50
Affected side (dominant:nondominant)^*^	79:35
Symptom duration (months)	11 (3–108)
Initial functional scores	
DASH	60.2 ± 14.1
Pain NRS on ulnar provocation test	7 (3–10)
Degree of ulnar plus variance (mm)	4 (2–7)
Type of TFCC lesion (IIA/IIB/IIC/IID/IIE)	(9/21/34/21/29)
Presence of carpal or distal unla bone enhancement	51 (45%)
Arthritis of ulno-carpal or distal radio-ulnar joint	29 (25%)

Values are expressed with mean ± SD/(range) or number of cases (proportion [%]). *In case of bilateral involvement, the more severely affected side was chosen for comparative analysis.

DASH = Disability of the Arm, Shoulder, and Hand score, TFCC = triangular fibrocartilage complex.

All patients had been initially treated with a short arm cast for four weeks followed by a removable short arm orthosis for about two weeks or so as required by the patient. Then, patients received formal physiotherapy with progressive mobility and strengthening exercises for six weeks and offered oral nonsteroidal anti-inflammatory drugs. The frequency of physiotherapy was typically twice per week, but it varied by patient. Patients were also instructed to modify their lifestyle, limiting aggravating movements such as pronation, gripping, and ulnar deviation of the wrist for three months.

**Figure 1:**
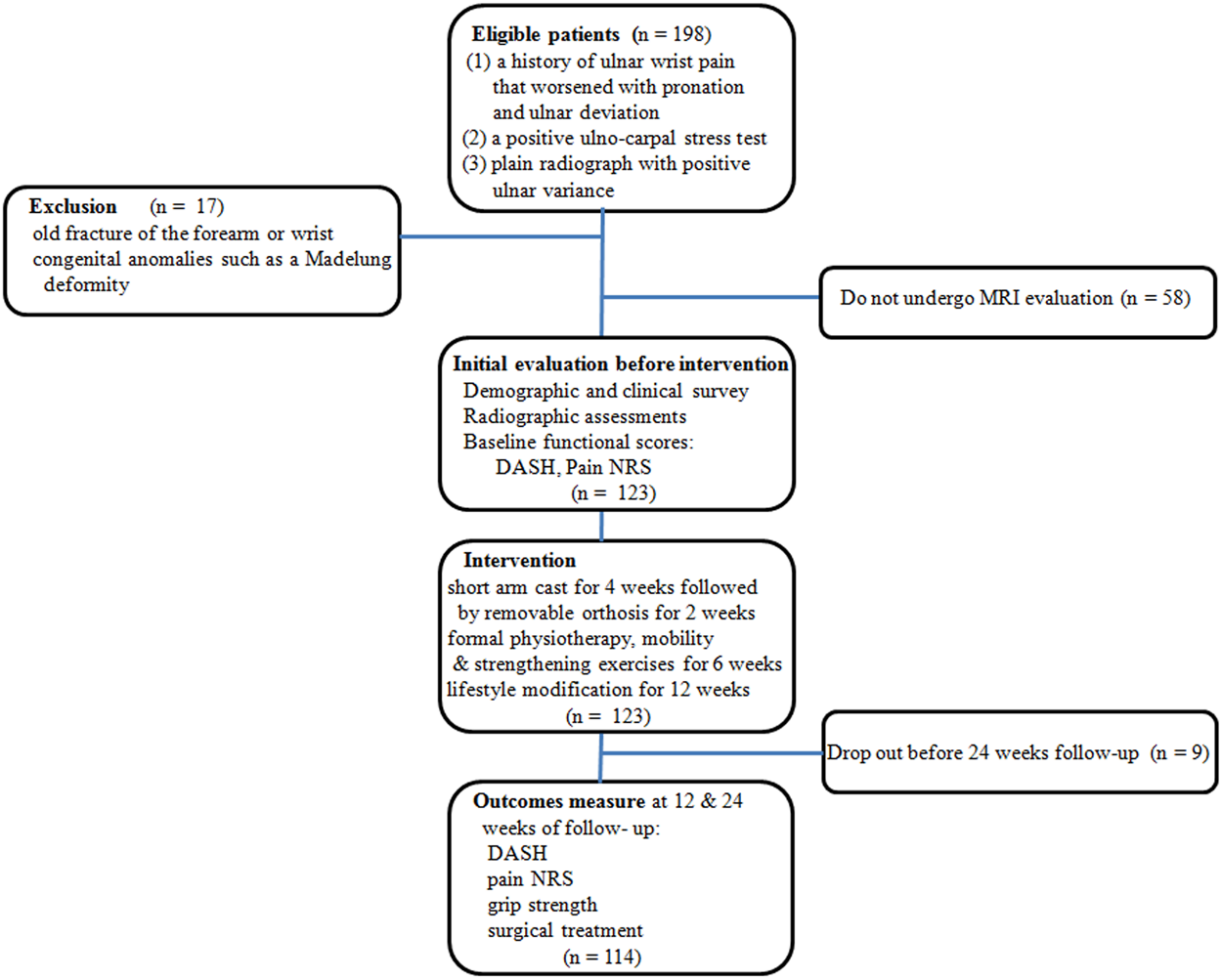
Flow diagram of study protocols.

Patients returned for their functional assessments at 12 (11–14) and 24 (22–26) weeks after conservative interventions (Fig. 1). We measured maximum pain-free grip strength in kilogram-force using a Jamar dynamometer (Asimow Engineering, Los Angeles, CA, USA) with the shoulder and forearm in neutral rotation; the dynamometer was calibrated according to a standard procedure by using standardized test weights, and we calculated the means for three measurements and used these means in the analyses. We also assessed pain severity on ulno-carpal stress test^11^ using an 11-point numeric rating scale (NRS), with 0 indicating no pain and 10 indicating the worst pain imaginable. We also analyzed patients’ self-reported outcomes with the Disabilities of the Arm, Shoulder and Hand (DASH) score; the DASH is a self-administered, upper-extremity specific questionnaire that consists of 30 items on physical functions, social-role function, symptoms, work, sleep, and confidence^12^. The items are rated on a five-point Likert-type scale where 1 is without difficulty or no symptom and 5 is unable to engage in activity or very severe symptoms. Thus, the DASH provides a best possible score of 0 and a worst possible score of 100. The DASH is user friendly, reliable, and valid for a range of upper extremity disorders^13,14^. A study investigator who was a trained nurse checked all returned questionnaires for completion, and the participants were assisted in completing the missing items.

We defined treatment failure, the primary outcome of this study, as persistence of pain (NRS score greater than 5) with positive ulno-carpal stress test or surgical intervention at six- month follow-up with conservative treatment. The decision to undergo surgery was mutual between patient and surgeon.

### Statistical Analysis

A post hoc power analysis indicated that the sample consisting of 114 patients would provide 90% statistical power with an α of 0.05 for a medium effect size (F^2^ of 0.15) for a regression with five main predictors.

We calculated descriptive statistics to determine the patients’ demographic, clinical, and radiographic characteristics, in particular, the relationships between potential predictors (demographic or clinical factors: age, gender, hand dominance, education level, work level, symptom duration, initial DASH and pain NRS scores; radiographic factors: degree of ulnar plus variance, TFCC lesion, presence of carpal or distal ulna bone enhancement, arthritis of ulno-carpal or distal radio-ulnar joint) and the outcome variables (pain NRS scores on ulnar provocation test greater than 5 or surgical treatment). We tested these relationships using an independent t test or a one-way ANOVA for categorical potential predictors and a correlation coefficient for continuous predictor variables. We selected bivariate predictors with p < 0.1 on bivariate analysis as candidates for the multivariable linear regression model to prevent model over-fitting, dummy coding the categorical variables and considering the largest subgroup to be the reference group.

### Ethical approval

All procedures performed in studies involving human participants were in accordance with the ethical standards of the institutional and/or national research committee and with the 1964 Helsinki declaration and its later amendments or comparable ethical standards.

## Results

**Figure 2:**
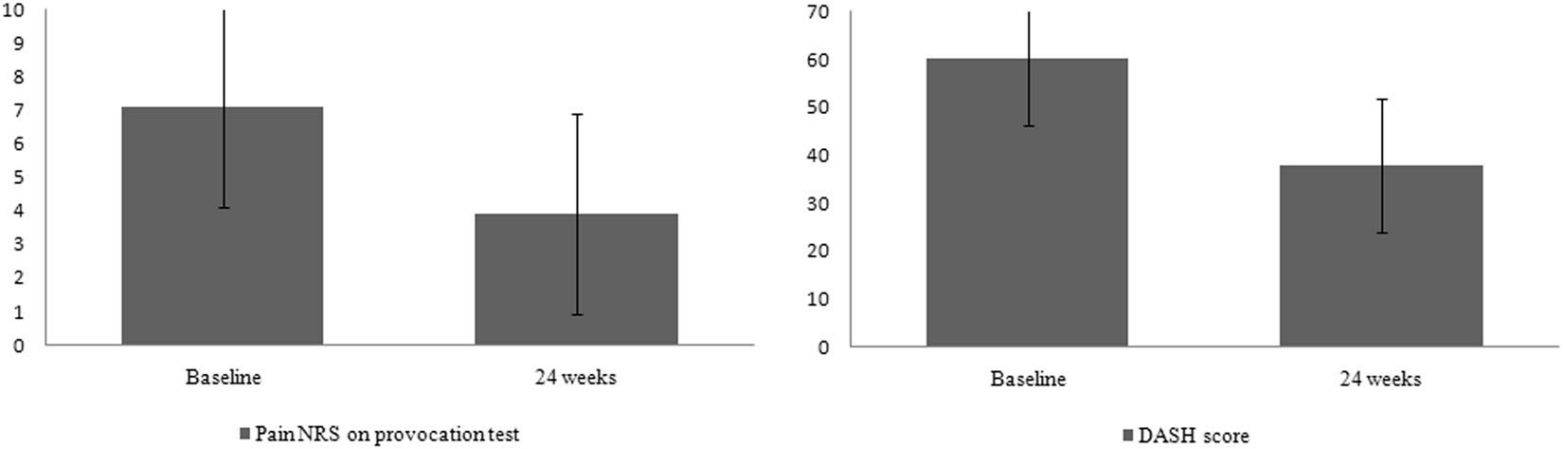
The mean pain NRS (A) and DASH (B) scores exhibited significant clinical improvement at the 24 week follow-up. NRS = numeric rating score, DASH = Disability of the Arm, Shoulder, and Hand score.

The mean pain NRS (7.1 ± 3.7 to 3.9 ± 3.0, p < 0.01) and DASH (60.2 ± 14.1 to 37.9 ± 13.9, p < 0.01) scores exhibited significant clinical improvement at the 24 week follow-up (Fig. 2). For the 24-week follow-up, 29 patients (25%) underwent ulnar shortening osteotomy due to persistent symptoms after conservative treatment. At the 24-week follow-up, 18 (16%) patients had pain NRS scores of greater than 5 on the ulnar provocation test, but they had not undergone surgery. Thus, the proportion of treatment failure was 47 (41%) after 24-week follow-up.

Patients with signal enhancement of the carpal or distal ulna on MRI had significantly higher pain NRS and DASH scores but similar grip strength to those without the MRI findings. However, increased ulnar puls variance, type of TFCC lesion, and arthritis of ulno- carpal or radio-ulnar joint were not associated with initial pain NRS score, grip strength, or DASH scores (Table 2). Grip strength was negatively correlated with age (r = −0.43, p = 0.02) and significantly higher in male and dominant side than female or non-dominant side (p = 0.01 and 0.04, respectively).

**Table 2:** Bivariate relationship analysis between radiologic findings and initial clinical features.

Variables	Pain NRS on provocation test	DASH scores	Grip strength
	Significance (*P*, t-test or ANOVA)		
Type of TFCC lesion	NS	NS	NS
Presence of carpal or distal unla enhancement	0.01	0.02	NS
Arthritis of ulno-carpal or distal radio-ulnar joint	NS	NS	NS
Degree of ulnar plus variance	Significance (*P*, Pearson correlation test)		
	NS	NS	NS

NRS = numeric rating scale, DASH = Disability of the Arm, Shoulder, and Hand score, TFCC = triangular fibrocartilage complex.

**Figure 3:**
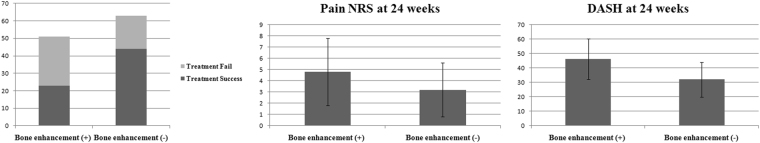
Comparisons of clinical outcomes in patients with and without bone enhancement on MRI; (A) treatment failure, (B) pain NRS, and (C) DASH score, NRS = numeric rating score, DASH = Disability of the Arm, Shoulder, and Hand score.

The treatment failure group had significantly higher initial pain scores on the ulnar provocation test, higher DASH scores, and longer symptom duration (p < 0.01, < 0.01, and 0.02, respectively, Table 3) but similar grip strength. In terms of the demographic factors, female sex was significantly different between the two groups (p = 0.03, Table 3); however, patient characteristics such as age, education level, and manual labor were not significant factors. In terms of the radiologic findings, presence of enhancement in the carpal or distal ulna bone on MRI differed significantly between the two groups (p = 0.01, Fig. 3), but static or dynamic ulnar variances, arthritic change, and the type of TFCC lesion were not significant factors.

**Table 3:** Clinical and radiologic differences between treatment success and failure groups.

Characteristics	Treatment success Group (n = 67)	Treatment failure Group (n = 47)	p value
Age (years)	39 ± 12	41 ± 13	0.40
Sex (female/male)	45/22	40/7	**0.03**
Body mass index	26.1 ± 3.9	27.1 ± 4.1	0.20
Less than a high school education	16 (24%)	9 (19%)	0.55
Manual labor	13 (19%)	10 (21%)	0.80
Dominant side	46 (61%)	33 (59%)	0.86
Symptom duration	9 ± 7	13 ± 10	**0.02**
Initial DASH	55 ± 16	67 ± 18	<**0.01**
Initial Pain NRS on provocation test	6 ± 3	8 ± 3	<**0.01**
Degree of ulnar plus variance	3.8 ± 1.5	4.3 ± 1.6	0.10
Type TFCC lesion (IIA/IIB/IIC/IID/IIE)	7/13/19/12/16	2/8/15/9/13	0.068
Presence of carpal or distal unla bone enhancement	23 (34%)	28 (60%)	**0.01**
Arthritis of ulno-carpal or distal radio-ulnar joint	15 (22%)	14(30%)	0.65

Values are expressed with mean ± SDs or number of cases (proportion); DASH = Disability of the Arm, Shoulder, and Hand score, TFCC = triangular fibrocartilage complex.

The results of multivariable logistic regression analysis indicated that female gender (OR = 1.39 [95% CI 1.28, 1.92], p = 0.02), symptom duration (OR = 1.27 [95% CI 1.10, 1.62], p = 0.03), high pain NRS score on ulnar provocation test (OR = 1.45 [95% CI 1.30, 2.32], p = 0.01), and enhanced carpal or distal ulna bone (OR = 1.82 [95% CI 1.51, 2.92], p = 0.01) were associated with a higher likelihood of treatment failure.

## Discussion

In cases of idiopathic ulnar impaction syndrome, nonoperative treatment should be provided initially because not all cases of radiographic ulnar impaction are symptomatic, and it is crucial to identify predictive factors related to the clinical symptoms and prognoses of UIS; however, few reports suggest specific clinical and radiologic features as predictive factors for conservative treatment of idiopathic UIS. The current study was an attempt to determine which preoperative clinical or radiologic features were associated with poor outcomes of conservative treatment for UIS. We found that female gender, long symptom duration, high pain NRS score on ulnar provocation test, and enhancement of carpal or distal ulna bone on MRI were associated with a higher likelihood of treatment failure after conservative treatment for idiopathic UIS.

In terms of patient demographic or clinical factors, female gender, longer symptom duration, and initial high pain NRS score on ulnar provocation test were associated with a higher likelihood of treatment failure after conservative management of idiopathic UIS. The natural history of ulno-carpal abutment is thought to be one of persistent or progressive derangement, and a long persistence of symptoms may be attributable to pathologic change to the ulno-carpal joint^7^. Long symptom duration was also reported to be associated with poor outcomes after ulnar shortening osteotomy^15^. The sex difference in treatment outcomes could in part be explained by higher physical vulnerability^16^ or pain sensitivity^17^ in women; musculoskeletal pain and disability were found to be more prevalent^18^ and more severe in women^19^. The ulno-carpal stress test is reportedly highly sensitive but not specific for UIS; other ulno-capal pathology including lunotriquetral ligament injury or isolated ulno-carpal arthritis will produce positive findings^20,21^. However, our results suggests that pain NRS scores on ulno-carpal stress test in the presence of a history of ulnar wrist pain worsened by pronation or ulnar deviation of the wrist and plain radiograph with positive ulnar variance had significant prognostic value for conservative treatment of idiopathic UIS.

In terms of radiologic factors, patients with the signal enhancement of carpal or distal ulna on MRI had higher pain NRS and DASH scores, and this was the only significant prognostic factor; almost none of the radiologic factors including degree of ulnar plus variance, type of TFCC lesion, or arthritis of ulno-carpal or distal radio-ulnar joint had a significant effect on the treatment failure for idiopathic UIS. These results are consistent with previous findings that diagnostic MRI findings and values are limited in TFCC injury^20^, suggesting that the diagnosis of UIS is made on clinical ground and supported by radiographic studies. However, MRI findings are often a precursor to plain radiographic findings^22^ and our results suggest that carpal or distal ulna bone enhancement is distinctive and helps to predict the prognosis. Subchondral bone marrow edema is an indirect sign of chondromalacia and an early UIS finding^22^. One previous study showed that the signal intensity often returns to normal after ulnar-shortening osteotomy^6^.

There were a number of limitations to our study. There were no follow-up data on functional outcomes beyond 24 weeks of conservative treatment. Some patients whose symptoms recurred likely had surgery whereas others could have exhibited improvement in objective findings and outcome scores after the study time period. In addition, there is no consensus about the best way of conservative management for UIS although several conservative interventions are routinely used in clinical practice^6,23,24^. The conservative treatment protocols with 3- to 4-month duration might be not sufficient for patients with a long symptom duration or bone enhancement on MRI. Second, surgical management is not a precise indicator of the efficacy of conservative treatment because the decision for surgery is multifactorial and not determined entirely by symptom abatement via treatment. Thus, we defined treatment failure as persistence of pain NRS score greater than 5 with positive ulno-carpal stress test result or surgical intervention at six-month follow-up. Furthermore, we only used one questionnaire to evaluate the patients’ functional outcomes, but the minimal clinically important differences or responses after conservative treatment for idiopathic UIS could differ across functional assessments, and another functional assessment might have resulted in different conclusions. Third, nine (7%) patients were lost to follow-up before the six-month evaluation, and there were some missing responses and questionnaires in our cohort. Finally, our subjects were limited to one ethnic population drawn from an urban area, and therefore, their characteristics and the study results may not be representative of general populations.

## Conclusion

After 24-week follow-up, approximately 60% of patients showed improvements in both objective and patient-reported outcomes after conservative treatment for idiopathic UIS. However, 25% patients underwent ulnar shortening osteotomy due to persistent symptoms after conservative treatment, and 16% patients who had not undergone surgery had pain NRS scores of greater than 5 on ulnocarpal provocation test at the last follow-up. Our study suggests that female gender, long symptom duration, initial high pain NRS score on ulnar provocation test, and enhancement of carpal or distal ulna bone on MRI were associated with a higher likelihood of treatment failure. Although the outcomes are not modifiable, knowledge of the factors that affect unsatisfactory outcomes offer physicians insight into predicting prognoses and interpreting treatment outcomes and can help patients set realistic expectations and increase satisfaction.

## References

[1] Sachar K (2008). Ulnar-sided wrist pain: evaluation and treatment of triangular fibrocartilage complex tears, ulnocarpal impaction syndrome, and lunotriquetral ligament tears. J Hand Surg Am.

[2] Tomaino MM, Elfar J (2005). Ulnar impaction syndrome. Hand Clin.

[3] Baek GH (2005). Ulnar shortening osteotomy in idiopathic ulnar impaction syndrome. J Bone Joint Surg Am.

[4] Katz DI, Seiler JG, Bond TC (2010). The treatment of ulnar impaction syndrome: a systematic review of the literature. J Surg Orthop Adv.

[5] Stockton DJ, Pelletier ME, Pike JM (2015). Operative treatment of ulnar impaction syndrome: a systematic review. J Hand Surg Eur Vol.

[6] Sammer DM, Rizzo M (2010). Ulnar impaction. Hand Clin.

[7] Jarrett CD, Baratz ME (2012). The management of ulnocarpal abutment and degenerative triangular fibrocartilage complex tears in the competitive athlete. Hand Clin.

[8] Imaeda T, Nakamura R, Shionoya K, Makino N (1996). Ulnar impaction syndrome: MR imaging findings. Radiology.

[9] Ersoy H, Pomeranz SJP (2015). Classification and Magnetic Resonance Imaging Findings of Ulnocarpal Impingement. J Surg Orthop Adv.

[10] Cerezal L (2002). Imaging findings in ulnar-sided wrist impaction syndromes. Radiographics.

[11] Nakamura R (1997). The ulnocarpal stress test in the diagnosis of ulnar-sided wrist pain. J Hand Surg Br.

[12] Hudak PL, Amadio PC, Bombardier C (1996). Development of an upper extremity outcome measure: the DASH (disabilities of the arm, shoulder and hand) [corrected]. The Upper Extremity Collaborative Group (UECG). Am J Ind Med.

[13] Szabo RM (2001). Outcomes assessment in hand surgery: when are they meaningful?. J Hand Surg Am.

[14] Gummesson C, Atroshi I, Ekdahl C (2003). The disabilities of the arm, shoulder and hand (DASH) outcome questionnaire: longitudinal construct validity and measuring self-rated health change after surgery. BMC Musculoskelet Disord.

[15] Iwasaki N, Ishikawa J, Kato H, Minami M, Minami A (2007). Factors affecting results of ulnar shortening for ulnar impaction syndrome. Clin Orthop Relat Res.

[16] Wijnhoven HA, de Vet HC, Picavet HS (2006). Prevalence of musculoskeletal disorders is systematically higher in women than in men. Clin J Pain.

[17] Wolfe F, Ross K, Anderson J, Russell IJ (1995). Aspects of fibromyalgia in the general population: sex, pain threshold, and fibromyalgia symptoms. J Rheumatol.

[18] Leveille SG, Zhang Y, McMullen W, Kelly-Hayes M, Felson DT (2005). Sex differences in musculoskeletal pain in older adults. Pain.

[19] Bingefors K, Isacson D (2004). Epidemiology, co-morbidity, and impact on health-related quality of life of self-reported headache and musculoskeletal pain–a gender perspective. Eur J Pain.

[20] Schmauss D (2016). Clinical tests and magnetic resonance imaging have limited diagnostic value for triangular fibrocartilaginous complex lesions. Arch Orthop Trauma Surg.

[21] Valdes K, LaStayo P (2013). The value of provocative tests for the wrist and elbow: a literature review. J Hand Ther.

[22] Cerezal L, del Pinal F, Abascal F (2004). MR imaging findings in ulnar-sided wrist impaction syndromes. Magn Reson Imaging Clin N Am.

[23] Sachar K (2012). Ulnar-sided wrist pain: evaluation and treatment of triangular fibrocartilage complex tears, ulnocarpal impaction syndrome, and lunotriquetral ligament tears. J Hand Surg Am.

[24] Ikeda M (2015). Conservative treatment using a newly designed custom-made wrist splint for ulnocarpal abutment syndrome. Prosthet Orthot Int.

